# Sequential Strategy Including FFR_CT_ Plus Stress-CTP Impacts on Management of Patients with Stable Chest Pain: The Stress-CTP RIPCORD Study

**DOI:** 10.3390/jcm9072147

**Published:** 2020-07-08

**Authors:** Andrea Baggiano, Laura Fusini, Alberico Del Torto, Patrizia Vivona, Marco Guglielmo, Giuseppe Muscogiuri, Margherita Soldi, Chiara Martini, Enrico Fraschini, Mark G. Rabbat, Francesca Baessato, Gloria Cicala, Maria L. Danza, Annachiara Cavaliere, Antonella Loffreno, Vitanio Palmisano, Francesca Ricci, Giulia Rizzon, Elisabetta Tonet, Giacomo M. Viani, Saima Mushtaq, Edoardo Conte, Andrea D. Annoni, Alberto Formenti, Maria E. Mancini, Franco Fabbiocchi, Piero Montorsi, Daniela Trabattoni, Alexia Rossi, Fabio Fazzari, Nicola Gaibazzi, Daniele Andreini, Emilio M. Assanelli, Antonio L. Bartorelli, Mauro Pepi, Andrea I. Guaricci, Gianluca Pontone

**Affiliations:** 1 Centro Cardiologico Monzino IRCCS, 20138 Milan, Italy; andrea.baggiano@cardiologicomonzino.it (A.B.); laura.fusini@ccfm.it (L.F.); alberico.deltorto@ccfm.it (A.D.T.); patrizia.vivona28@gmail.com (P.V.); marco.guglielmo@ccfm.it (M.G.); giuseppe.muscogiuri@ccfm.it (G.M.); margherita.soldi@ccfm.it (M.S.); enrico.fraschini@ccfm.it (E.F.); giacomo.viani@gmail.com (G.M.V.); saima.mushtaq@ccfm.it (S.M.); edoardo.conte@ccfm.it (E.C.); andrea.annoni@ccfm.it (A.D.A.); alberto.formenti@ccfm.it (A.F.); maria.mancini@ccfm.it (M.E.M.); franco.fabbiocchi@ccfm.it (F.F.); piero.montorsi@ccfm.it (P.M.); daniela.trabattoni@ccfm.it (D.T.); daniele.andreini@ccfm.it (D.A.); emilio.assanelli@ccfm.it (E.M.A.); antonio.bartorelli@ccfm.it (A.L.B.); mauro.pepi@ccfm.it (M.P.); 2 Department of Clinical Sciences and Community Health, Cardiovascular Section, University of Milan, 20122 Milan, Italy; 3 Department of Radiology, Azienda Ospedaliero-Universitaria, 43126 Parma, Italy; chiaramartini10@gmail.com; 4 Loyola University Medical Center, Center for Heart & Vascular Medicine, Maywood, IL 60153, USA; mrabbat@lumc.edu; 5 Edward Hines Jr. VA Hospital, Hines, IL 60141, USA; 6 Divisione di Cardiologia, Dipartimento di Medicina, Università degli Studi, 37134 Verona, Italy; francesca.baessato89@gmail.com; 7 Dipartimento di Medicina e Chirurgia, Azienda Ospedaliero-Universitaria di Parma, Università degli Studi, 43126 Parma, Italy; gloria.cicala@live.com; 8 Division of Cardiology, University of Rome Tor Vergata, 00133 Rome, Italy; ludovicadanza@gmail.com; 9 Dipartimento di Medicina, Istituto di Radiologia, Università degli Studi, 35128 Padova, Italy; annachiara88cavaliere@gmail.com (A.C.); giulia.rizzon@hotmail.it (G.R.); 10 U.O.C. Cardiologia 1, Ospedale di Circolo e Fondazione Macchi, Università degli Studi, 21100 Varese, Italy; antonella.loffreno@gmail.com; 11 Diagnostica per Immagini, Università degli Studi di Cagliari, Monserrato, 09042 Cagliari, Italy; vitanio88@gmail.com; 12 Department of Biomedicine and Prevention Division of Diagnostic Imaging University of Rome Tor Vergata, 00133 Rome, Italy; francesca.ricci3@studenti.unipr.it; 13 Cardiovascular Institute, Azienda Ospedaliero-Universitaria di Ferrara, Cona, 44124 Ferrara, Italy; tonet.elisabetta@gmail.com; 14 Department of Biomedical Sciences, Humanitas University, Pieve Emanuele, 20090 Milan, Italy; alexia.rossi@hunimed.eu; 15 Department of Diagnostic Imaging, Humanitas Research Hospital, Rozzano, 20089 Milan, Italy; fabio.fazzari@humanitas.it; 16 Unità Operativa di Cardiologia, Azienda Ospedaliero-Universitaria di Parma, Università degli Studi, 43126 Parma, Italy; ngaibazzi@gmail.com; 17 Department of Biomedical and Clinical Sciences “Luigi Sacco”, University of Milan, 20157 Milan, Italy; 18 Institute of Cardiovascular Disease, Department of Emergency and Organ Transplantation, University Hospital Policlinico of Bari, 70124 Bari, Italy; andrea.guaricci@gmail.com

**Keywords:** clinical management, computed tomography, coronary artery disease, fractional flow reserve, myocardial perfusion

## Abstract

Stress computed tomography perfusion (Stress-CTP) and computed tomography-derived fractional flow reserve (FFR_CT_) are functional techniques that can be added to coronary computed tomography angiography (cCTA) to improve the management of patients with suspected coronary artery disease (CAD). This retrospective analysis from the PERFECTION study aims to assess the impact of their availability on the management of patients with suspected CAD scheduled for invasive coronary angiography (ICA) and invasive FFR. The management plan was defined as optimal medical therapy (OMT) or revascularization and was recorded for the following strategies: cCTA alone, cCTA+FFR_CT_, cCTA+Stress-CTP and cCTA+FFR_CT_+Stress-CTP. In 291 prospectively enrolled patients, cCTA+FFR_CT_, cCTA+Stress-CTP and cCTA+FFR_CT_+Stress-CTP showed a similar rate of reclassification of cCTA findings when FFR_CT_ and Stress-CTP were added to cCTA. cCTA, cCTA+FFR_CT_, cCTA+Stress-CTP and cCTA+FFR_CT_+Stress-CTP showed a rate of agreement versus the final therapeutic decision of 63%, 71%, 89%, 84% (cCTA+Stress-CTP and cCTA+FFR_CT_+Stress-CTP vs cCTA and cCTA+FFR_CT_: *p* < 0.01), respectively, and a rate of agreement in terms of the vessels to be revascularized of 57%, 64%, 74%, 71% (cCTA+Stress-CTP and cCTA+FFR_CT_+Stress-CTP vs cCTA and cCTA+FFR_CT_: *p* < 0.01), respectively, with an effective radiation dose (ED) of 2.9 ± 1.3 mSv, 2.9 ± 1.3 mSv, 5.9 ± 2.7 mSv, and 3.1 ± 2.1 mSv. The addition of FFR_CT_ and Stress-CTP improved therapeutic decision-making compared to cCTA alone, and a sequential strategy with cCTA+FFR_CT_+Stress-CTP represents the best compromise in terms of clinical impact and radiation exposure.

## 1. Introduction

Coronary computed tomography angiography (cCTA) was introduced as an anatomic imaging method to rule out the presence of coronary artery disease (CAD) [1] and also for improving prognostic assessment beyond baseline risk factor evaluation and functional stress test findings [2,3,4]. However, several factors limit its specificity and positive predictive value [5,6]. In this regard, there is an emerging body of literature on the added value of stress myocardial perfusion utilizing computed tomography (Stress-CTP) [7,8,9,10,11,12] and computed tomography-derived fractional flow reserve (FFR_CT_) on top of cCTA [9,13,14]. Moreover, several studies have compared the diagnostic accuracy of these novel CT functional techniques and they have shown favorable and comparable results, with increased diagnostic performance in detecting flow-limiting stenoses when compared to cCTA alone [15,16]. However, no comparison between the influence of FFR_CT_ and Stress-CTP on the management of patients has been performed [17,18]. Therefore, the aim of the study is to evaluate the allocation of patients to optimal medical treatment (OMT) or revascularization using cCTA, cCTA+FFR_CT_ and cCTA+Stress-CTP, and the rate of agreement in terms of the vessels to be revascularized in relation to invasive coronary angiography (ICA) and FFR-defined significance.

## 2. Experimental Methods

The retrospective analysis of this study was conducted on subjects prospectively enrolled in the PERFECTION Study, a longitudinal prospective study with consecutive cohorts designed to compare the feasibility and accuracy of integrated cCTA+FFR_CT_ versus cCTA+Stress-CTP for the diagnosis of functionally significant CAD [19]. This study complied with the Declaration of Helsinki. Moreover, the institutional ethical study committee approved the protocol (R250/15-CCM 262), and all patients meeting the selection criteria were asked to sign an informed consent. A structured interview was performed to collect a clinical history and cardiac risk factors.

Two consecutive cohorts of patients with a pre-test likelihood of CAD > 50% according to the updated Diamond-Forrester risk model score were evaluated. The first cohort of 147 patients were evaluated with the Static Stress-CTP, while the second cohort of 144 patients were tested with the Dynamic Stress-CTP. The first cohort of patients was screened with consecutive symptomatic patients with suspected CAD referred for non-emergent, clinically indicated ICA between October 2015 and May 2017, while the second cohort was screened following the same criteria between June 2017 and April 2019.

**Figure A1:**
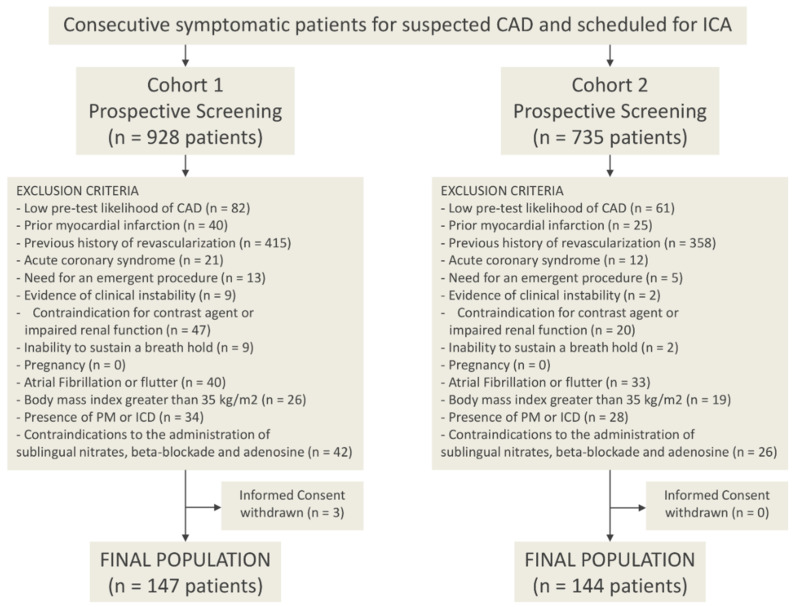
Study flow diagram. Consecutive symptomatic patients referred for suspected CAD and scheduled for clinically indicated ICA were screened. After the application of inclusion and exclusion criteria, 291 patients were enrolled, 147 in the first arm, in which Static Stress-CTP was performed, and 144 in the second arm, in which Dynamic Stress-CTP was performed. CAD: coronary artery disease; ICA: invasive coronary angiography; Stress-CTP: stress computed tomography perfusion.

**Figure A2:**
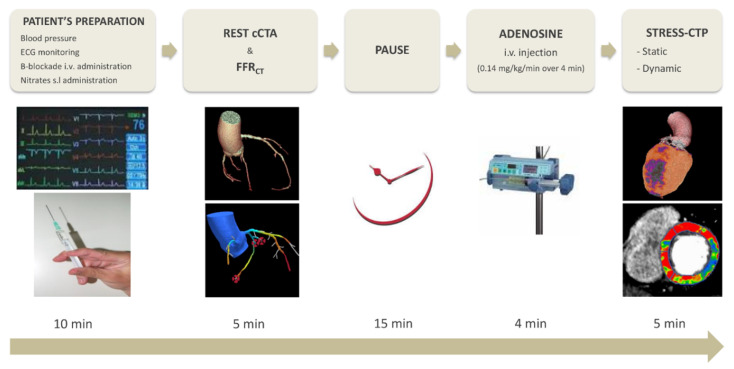
Study protocol. Patients were asked to refrain from smoking and caffeine for 24 h and to maintain fasting for 6 h before the scan. All patients were treated with sublingual nitrates to ensure vasodilatation. In patients with a resting HR > 65 beats/min, metoprolol was intravenously administered, with a titration dose up to 15 mg to achieve a target HR ≤ 65 beats/min. However, all patients were studied even if the target HR was not reached. cCTA and FFR_CT_ were evaluated with the acquisition of the first dataset; then, after a period of fifteen minutes, perfusion evaluation was performed during adenosine administration with the acquisition of the second dataset (Static or Dynamic protocols were used according to the specific arm of the study). HR: heart rate; cCTA: coronary computed tomography angiography; FFR_CT_: computed tomography-derived fractional flow reserve.

The study flow diagram and the study protocol are shown in Appendix A (Figure A1 and Figure A2, respectively).

Appendix B details the methods used to perform and interpret the results of the studied techniques, including Rest cCTA, FFR_CT_, Static Stress-CTP, Dynamic Stress-CTP, ICA and invasive FFR. The method used to calculate radiation exposure is also described.

**Figure 1:**
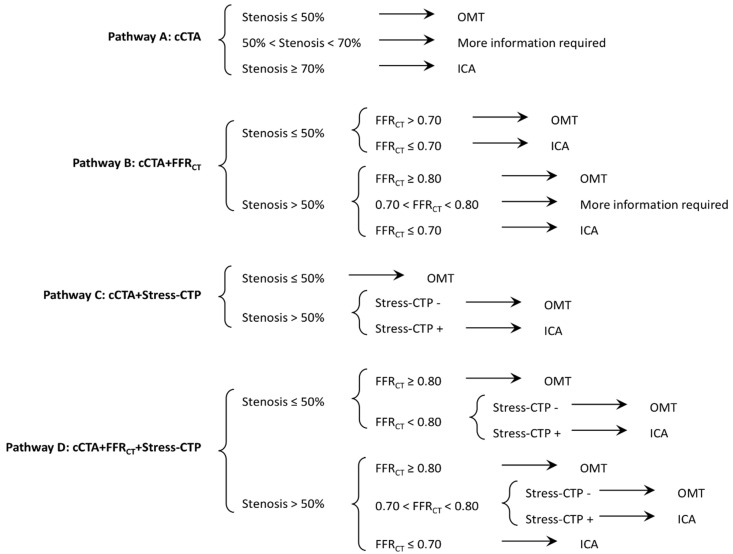
Clinical management algorithm. The clinical management algorithms to OMT, revascularization, or further information for cCTA alone (A), for the combined approach of cCTA+FFR_CT_ (B), for the combined approach of cCTA+Stress-CTP (C) and for the sequential approach of cCTA+FFR_CT_+Stress-CTP (D) are illustrated. OMT: optimal medical treatment; cCTA: coronary computed tomography angiography; OMT: optimal medical treatment; ICA: invasive coronary angiography; FFR_CT_: computed tomography-derived fractional flow reserve; Stress-CTP: stress computed tomography perfusion.

**Clinical management according to cCTA results only (Pathway A).** As a first step, the clinical management of patients according to cCTA results was assessed according to the criteria as shown in Figure 1: if all coronary stenoses were less than 50%, OMT was chosen; if at least one coronary stenosis was estimated in the range of 50% to 70% of vessel lumen reduction, more information was required; if at least one coronary stenosis resulted in more than 70% of vessel lumen reduction, invasive assessment was indicated.

**Clinical management according to addition of FFR_CT_ analysis to cCTA (Pathway B).** Clinical management when both cCTA and FFR_CT_ were available was investigated according to the following criteria (Figure 1): (1) in the case of stenoses ≤ 50%, OMT was prescribed with FFR_CT_ values ≥ 0.7 for all vessels, while ICA was indicated if FFR_CT_ was < 0.7 for at least one vessel; (2) in the case of cCTA with at least one stenosis > 50% of lumen reduction, if FFR_CT_ values were > 0.8 for all vessels, OMT was prescribed, while if FFR_CT_ was in the range of 0.7 to 0.8 for at least one vessel, more information was required; finally, if FFR_CT_ was less than 0.7 for at least one vessel, ICA was indicated.

**Clinical management according to addition of Stress-CTP results to cCTA (Pathway C).** Clinical management when both cCTA and Stress-CTP (where Static or Dynamic protocol was used) were available was investigated according to the following criteria (Figure 1): (1) in the case of coronary stenoses ≤ 50% of lumen reduction, OMT was prescribed regardless of the Stress-CTP results; (2) in the case of cCTA with at least one stenosis > 50% of lumen reduction, if perfusion defects were present, ICA was indicated, while in the absence of perfusion defects, OMT was prescribed.

**Clinical management according to addition of Stress-CTP on top of cCTA and FFR_CT_ analysis (Pathway D).** Clinical management when Stress-CTP was available on top of cCTA and FFR_CT_ analysis was investigated according to the following criteria (Figure 1). In the setting of cCTA ≤ 50%, we indicated: (a) OMT in the case of FFR_CT_ ≥ 0.8 or FFR_CT_ < 0.8 but with a negative matched Stress-CTP; and (b) ICA in the case of FFR_CT_ < 0.8 with a positive matched Stress-CTP. In the setting of cCTA > 50%, we indicated: (a) OMT in the case of FFR_CT_ ≥ 0.8 or FFR_CT_ between 0.7–0.8 but with a negative matched Stress-CTP; (b) ICA in the case of FFR_CT_ between 0.7–0.8 with a positive matched Stress-CTP or in the case of FFR_CT_ < 0.7, regardless of Stress-CTP findings.

**Endpoints.** The primary endpoint of the rate of agreement with the final management decision (defined as the ratio between the number of patients in which the therapeutic decision matched the final decision to the total number of patients) was measured for each strategy.

The following endpoints were also measured as part of a secondary analysis:

(a) Overall evaluability, defined as the ratio between the number of patients in which the strategy information was applied to the total number of patients
(b) Rate of reclassification, defined as the ratio between the number of patients who underwent a different management as compared to the initial decision to the total number of patients
(c) Rate of agreement on the vessels to be revascularized, defined as the ratio between the number of patients in which the vessel to be revascularized matched the final decision to the total number of patients
(d) Cumulative effective dose (ED), defined as the cumulative radiation exposure of the strategy.

**Statistical analysis.** Continuous variables were expressed as mean ± standard deviation (SD) and discrete variables as absolute numbers and percentages. Comparisons between each endpoint of all strategies were assessed with a χ^2^ test. A *p*-value < 0.05 was considered significant. Statistical analysis was performed with SPSS, version 25 software (SPSS Inc., Chicago, IL, USA) and R version 3.5.1.

## 3. Results

**Study population.**Table 1 shows the patients’ baseline characteristics. The study population consisted of 291 patients (Mean age: 65 ± 9 years; Male: 76%) with a prevalence of obstructive and functionally significant CAD at invasive evaluation of 67% and 49%, respectively.

**Figure 2:**
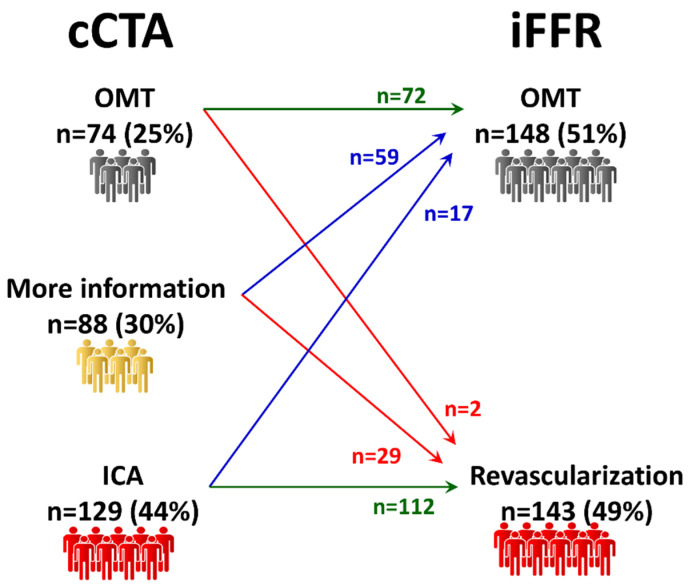
Management according to cCTA alone. Nearly one third of the population needed further assessment to be allocated to OMT or revascularization. cCTA: coronary computed tomography angiography; OMT: optimal medical treatment; ICA: invasive coronary angiography; iFFR: invasive fractional flow reserve.

**Management according to cCTA only.** Rest cCTA was successfully performed in all patients with a mean ED of 2.9 ± 1.3 mSv (Table 1). The overall evaluability was 92% (267 out of 291 patients). The clinical management according to cCTA alone is illustrated in Figure 2. Seventy-four (25%) patients were treated with OMT, 129 (44%) were sent to ICA, while 88 (30%) patients needed more information for a clinical decision. Once patients were sent to assessment with ICA and invasive FFR, the number of patients treated with OMT increased to 148 (51%), while the number of patients that were treated with revascularization increased to 143 (49%).

The rate of agreement with the final decision was 63% (184 out of 291 patients) and the rate of agreement of vessels to be revascularized was 57% (165 out of 291 patients).

**Figure 3:**
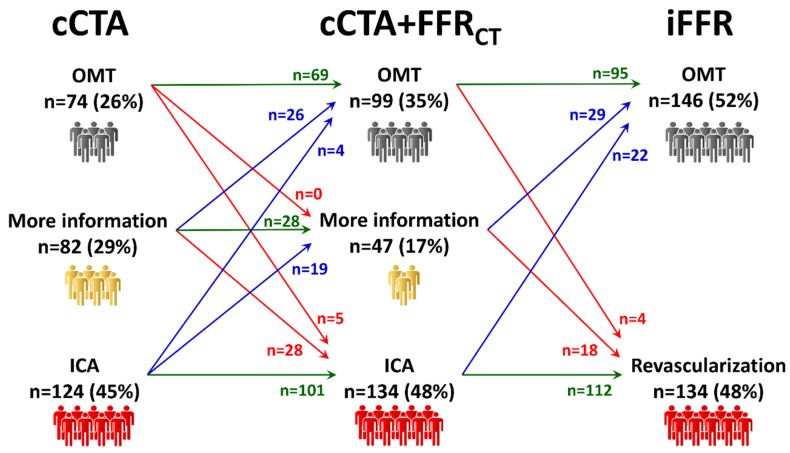
Management according to the addition of FFR_CT_ to cCTA. If FFR_CT_ is added to cCTA, there is a substantial equal redistribution of patients previously without a clear management indication to OMT or to revascularization. cCTA: coronary computed tomography angiography; OMT: optimal medical treatment; ICA: invasive coronary angiography; FFR_CT_: computed tomography-derived fractional flow reserve; iFFR: invasive fractional flow reserve.

**Management according to addition of FFR_CT_ to cCTA.** FFR_CT_ analysis was obtained in 280 out of 291 patients (96%) with an overall evaluability of the cCTA+FFR_CT_ strategy of 89% (258 out of 291 patients). The clinical management according to FFR_CT_ analysis on top of cCTA is shown in Figure 3. The addition of FFR_CT_ to cCTA increased the number of patients referred to OMT, from 74 (26%) to 99 (35%), and also increased direct referral to ICA, from 124 (45%) to 134 (48%). The “grey-zone”, in which more information was necessary for a clinical decision, was reduced from 82 (29%) to 47 (17%) patients. Once patients underwent invasive assessment, the number of patients treated with OMT increased to 146 (52%), while the number of patients treated with revascularization remained unchanged at 134 (48%). 

The rate of reclassification of patients with FFR_CT_ was 28% (82 out of 291 patients), the rate of agreement with the final treatment decision was 71% (207 out of 291 patients) and the rate of agreement on vessels to be revascularized was 63% (183 out of 291 patients).

**Management according to addition of Stress-CTP to cCTA.** Stress-CTP was performed in 287 out of 291 patients (99%) with an overall evaluability of the cCTA+Stress-CTP strategy of 90% (263 out of 291 patients). The mean ED of cCTA+Stress-CTP strategy was 5.9 ± 2.7 mSv.

**Figure 4:**
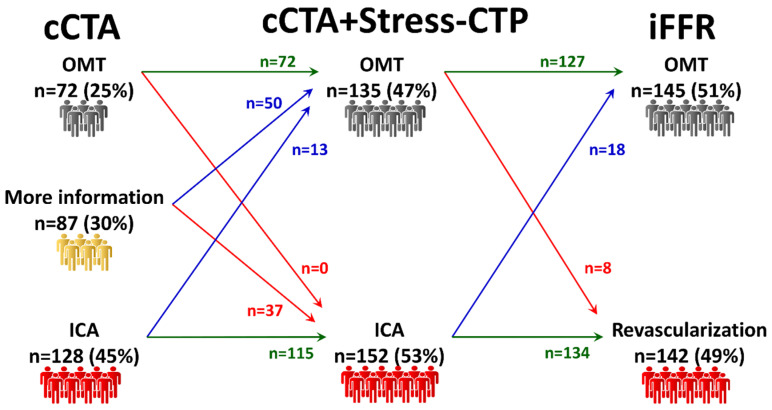
Management according to the addition of Stress-CTP to cCTA. If Stress-CTP is added to cCTA, more than two-thirds of patients who were previously without a clear management indication were allocated to OMT. cCTA: coronary computed tomography angiography; OMT: optimal medical treatment; ICA: invasive coronary angiography; Stress-CTP: stress computed tomography perfusion; iFFR: invasive fractional flow reserve.

The clinical management according to Stress-CTP analysis on top of cCTA is illustrated in Figure 4. The addition of Stress-CTP led to 87 (30%) patients who needed more information to decide on clinical management, with 50 patients treated with OMT, and 37 patients sent to ICA. After all patients were assessed with the invasive reference standard, the number of patients treated with OMT or revascularization changed minimally, with a mild increase in patients managed with OMT, and a proportional reduction in patients treated with revascularization. 

The rate of reclassification of patients with CTP was 34% (100 out of 291 patients), the rate of agreement with the final treatment decision was 89% (261 out of 291 patients) and the rate of agreement on vessels to be revascularized was 74% (216 out of 291 patients).

**Management according to sequential addition of FFR_CT_ and Stress-CTP to cCTA.** The sequential strategy was available in 254 patients, with an overall evaluability of 87%. The mean ED of the cCTA+FFR_CT_+Stress-CTP strategy was 3.5 ± 2.1 mSv.

**Figure 5:**
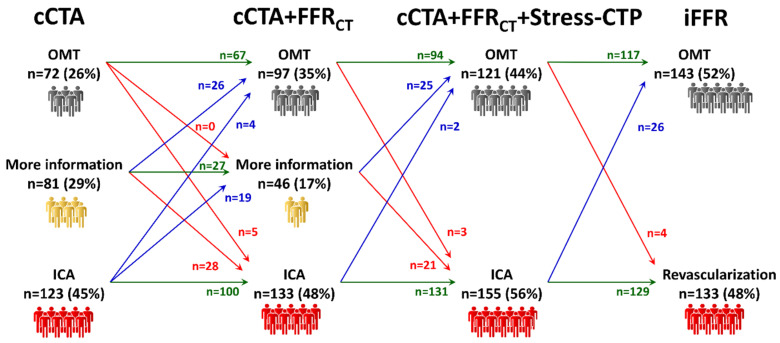
Management according to the sequential addition of FFR_CT_ and Stress-CTP to cCTA. If a sequential approach is used, patients without a clear management indication after FFR_CT_ analysis can be allocated to OMT or revascularization after Stress-CTP assessment. cCTA: coronary computed tomography angiography; OMT: optimal medical treatment; ICA: invasive coronary angiography; FFR_CT_: computed tomography-derived fractional flow reserve; Stress-CTP: stress computed tomography perfusion; iFFR: invasive fractional flow reserve.

The clinical management according to the sequential addition of FFR_CT_ and Stress-CTP to cCTA is displayed in Figure 5. The addition of functional tools led to a progressive increase in the number of patients treated with OMT from 72 (26%) with cCTA only to 97 (35%) when FFR_CT_ was added, and 121 (44%) with the addition of Stress-CTP. The number of patients in the “grey-zone” in the cCTA analysis was nearly halved from 81 to 46 patients by the addition of FFR_CT_, and this remaining population was further divided into two approximately equal parts with the addition of Stress-CTP. At the end of this sequential approach, 121 patients (44%) were managed with OMT while 155 (56%) were referred to ICA. After invasive assessment, the number of patients treated with OMT increased to 143 (52%), while patients treated with revascularization were reduced to 133 (48%). The rate of reclassification of patients was 37% (109 out of 291 patients), the rate of agreement with the final treatment decision was 84% (246 out of 291 patients) and the rate of agreement on vessels to be revascularized was 70% (205 out of 291 patients).

**Figure 6:**
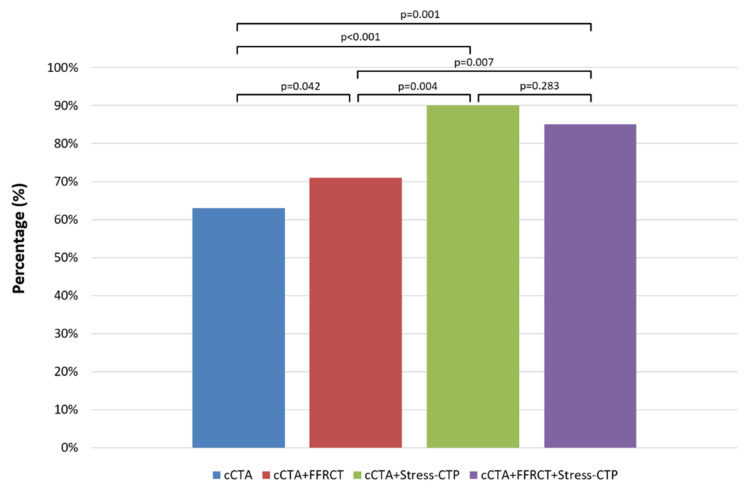
Primary endpoint of the study between different diagnostic strategies. Both the cCTA+Stress-CTP and cCTA+FFR_CT_+Stress-CTP strategies showed a higher rate of agreement with the final decision as compared to cCTA alone and the cCTA+FFR_CT_ strategy. cCTA: coronary computed tomography angiography; Stress-CTP: stress computed tomography perfusion; FFR_CT_: computed tomography-derived fractional flow reserve.

**Figure 7:**
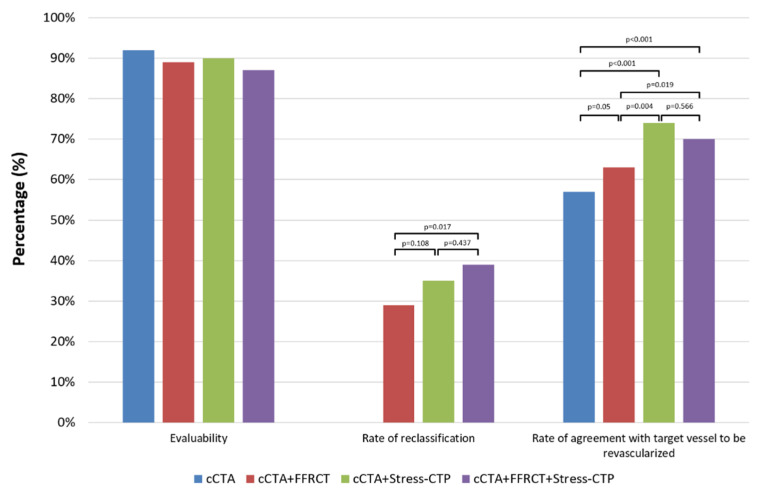
Secondary endpoints of the study between different diagnostic strategies. No differences were found in terms of evaluability between the different strategies. Both the cCTA+Stress-CTP and cCTA+FFR_CT_+Stress-CTP strategies showed a higher rate of patient reclassification and rate of agreement for the target vessel to be revascularized as compared to cCTA alone and the cCTA+FFR_CT_ strategy. cCTA: coronary computed tomography angiography; Stress-CTP: stress computed tomography perfusion; FFR_CT_: computed tomography-derived fractional flow reserve.

**Comparison between cCTA, cCTA+FFR_CT_, cCTA+Stress-CTP and the sequential strategy.**Figure 6 and Figure 7 show the comparison between the primary and secondary endpoints of different strategies, respectively. The rate of agreement with the final decision was significantly higher for cCTA+Stress-CTP and for cCTA+FFR_CT_+Stress-CTP versus other approaches (*p* < 0.01), with no difference between them. No differences were found in terms of evaluability between the different strategies. Both the cCTA+Stress-CTP and cCTA+FFR_CT_+Stress-CTP strategies showed a higher rate of patient reclassification and a higher rate of target vessels to be revascularized as compared to the cCTA+FFR_CT_ strategy.

**Figure 8:**
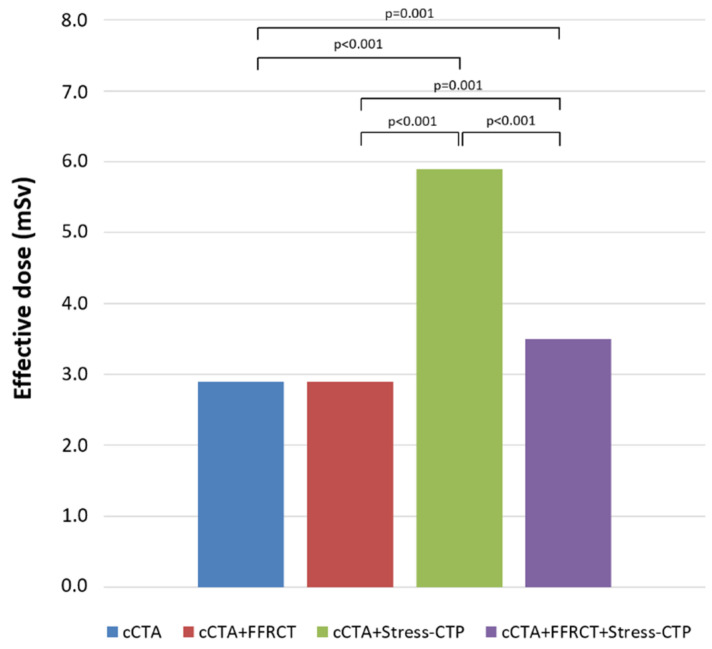
Radiation exposure. In terms of radiation exposure, the cCTA+Stress-CTP strategy was associated with the highest ED with a progressive reduction in the ED for cCTA+FFR_CT_+Stress-CTP and cCTA+FFR_CT_. cCTA: coronary computed tomography angiography; Stress-CTP: stress computed tomography perfusion, ED: effective dose; FFR_CT_: computed tomography-derived fractional flow reserve.

The overall radiation exposure according to each diagnostic strategy is reported in Figure 8. The cCTA+Stress-CTP strategy was associated with the highest ED with a progressive reduction in the ED for cCTA+FFR_CT_+Stress-CTP and cCTA+FFR_CT_ (*p* < 0.001).

**Figure 9:**
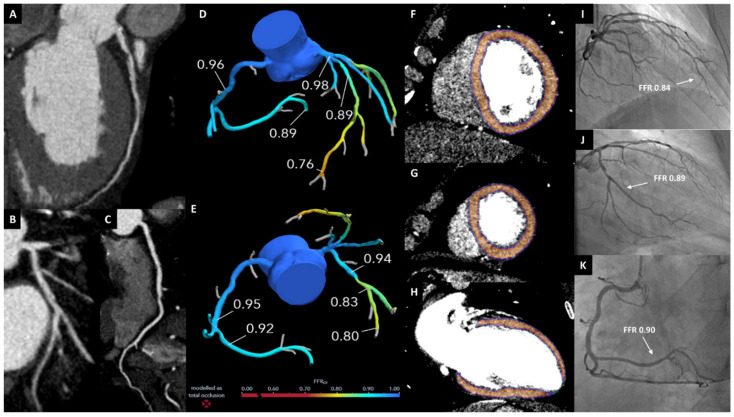
Clinical Case. A 54 y/o male with hypertension, dyslipidaemia and recent onset of atypical chest pain. Panels (**A**–**C**): cCTA showing significant stenosis in the proximal LAD (**A**), moderate stenosis in the proximal LCx (**B**), and moderate stenosis in the mid RCA (**C**). Panels (**D**,**E**): FFR_CT_ showing grey-zone values in the apical LAD, negative values in the RCA and LCx. Panels (**F**–**H**): Static Stress-CTP with short axis views (**F**,**G**) and 2-chamber long axis view (**H**) showing absence of perfusion defects. Panels (**I**–**K**): ICA showing moderate stenoses in the proximal LAD (**I**) and LCx (**J**), and mild stenosis in the mid RCA (**K**), all with negative FFR values. cCTA: coronary computed tomography angiography; LAD: left anterior descending; LCx: left circumflex; RCA: right coronary artery; FFR_CT_: computed tomography-derived fractional flow reserve; Stress-CTP: stress computed tomography perfusion; ICA: invasive coronary angiography; FFR: fractional flow reserve.

A representative case example is illustrated in Figure 9.

## 4. Discussion

The main findings of this study are (a) the addition of both FFR_CT_ and Stress-CTP on top of cCTA led to the reclassification of approximately one third of patients with intermediate to high risk for CAD; (b) compared to the cCTA+FFR_CT_ strategy, cCTA+Stress-CTP showed a better performance in terms of final therapeutic decision-making and target vessels to be revascularized, however, it is also associated with higher radiation exposure; and (c) a sequential strategy including cCTA+FFR_CT_+Stress-CTP where FFR_CT_ is the gatekeeper for the decision to perform a Stress-CTP, seems to provide the best balance between performance and radiation exposure. 

The first step in assessing CAD is the identification of coronary plaque, as the presence of coronary atherosclerosis is a crucial element in determining patients’ prognosis [4,20,21]. Unfortunately, anatomic assessment alone is often not enough to fully determine patient management, especially when patients with high a pre-test likelihood of CAD are evaluated. This is due to the increased coronary calcium burden and disease extension, which are factors that impair the ability of coronary cCTA to correctly rule out significant CAD [5].

Recently, there has been extensive data published regarding the role of FFR_CT_ in this clinical context. This tool is not only accurate in assessing for ischemia [22], but it has been shown to be very helpful in guiding patient management [13,23] in real-word experiences, such as in the ADVANCE Registry [9,24] and in Aarhus, Denmark [25]. Our study of 291 patients confirmed the increased discrimination of functionally relevant stenoses assessed by FFR_CT_, even in a population with a high burden of significant disease. Even though we used a wide “grey-zone”, between 0.7 and 0.8 in our study, this tool was able to reclassify two-thirds of the patients that required further assessment in order to receive a specific treatment. Moreover, compared to invasive assessment, FFR_CT_ is able to increase the overall diagnostic performance and correct pattern of treatment at both the patient and vessel-specific level.

There is less evidence to support the clinical role of Stress-CTP, and this paucity of data is mainly related to the relatively recent introduction of new generation scanners. Wong et al. used a 320-slice scanner to demonstrate that the combined approach of cCTA+Static Stress-CTP is superior to cCTA alone in assessing the presence of functionally relevant stenosis, using invasive FFR as a reference standard [26]. Using the same reference standard, Coenen et al. used dual energy scanners to show an increase in the diagnostic accuracy of a combined cCTA and Dynamic Stress-CTP approach compared to anatomical assessment alone (AUC 0.78 vs. 0.70) [16]. Finally, Pontone et al. used a last generation whole-heart scanner and showed that an integrated approach (cCTA+Static or Dynamic CTP) had better diagnostic accuracy in the assessment of patients with intermediate-to-high pre-test likelihood of CAD evaluated, again, using invasive FFR as the reference standard [15,27]. Importantly, it was possible to achieve acceptable radiation exposure (<6 mSv for cCTA+Static Stress-CTP and <9 mSv for cCTA+Dynamic Stress-CTP) as well as high diagnostic accuracy (AUC of 0.92 for cCTA+Static Stress-CTP and 0.88 for cCTA+Dynamic Stress-CTP in a per-vessel analysis).

Therefore, the question regarding which of these functional non-invasive techniques should be used, and in which patients is of great interest. Recently, we demonstrated that both Static [15] and Dynamic Stress-CTP [27] showed similar diagnostic performance as compared to invasive evaluation. However, a comparison between Stress-CTP and FFR_CT_ in terms of impact on clinical decision-making is not available. In a sub-study of the RIPCORD trial, Curzen et al. demonstrated that the availability of FFR_CT_ has a substantial effect on the ability to identify significant CAD, and therefore, on the management of patients with stable chest pain compared to cCTA alone [17]. This finding mimics the results found for invasive ICA and FFR in the RIPCORD study.

Our study not only confirmed the additional value of FFR_CT_ and Stress-CTP to cCTA alone, but also demonstrated that a sequential strategy including cCTA alone, followed by FFR_CT_ as a gatekeeper of Stress-CTP represents the best compromise in terms of clinical decision-making and radiation exposure. Due to the recent clinical application of this functional strategy, large-scale multi-center randomized trials addressing the cost-effectiveness and prognostic implications of CT myocardial perfusion are not yet available. To fill this gap in evidence, the CTP-PRO study, an international, multi-center, prospective, open-label, randomized controlled study evaluating the cost-effectiveness and the prognosis of a cCTA+Stress-CTP strategy versus usual care in intermediate-high risk patients with suspected or known CAD has been designed [28] and the results are eagerly awaited.

This study has limitations. Mainly, we used an existing dataset of patients in order to test our hypothesis, to deliver a retrospective analysis of a prospectively collected population, and therefore, the final number of subjects was estimated according to a predefined statistical power for other endpoints.

## 5. Conclusions

In conclusion, this retrospective analysis of the PERFECTION study demonstrates that the addition of functional assessment with FFR_CT_ and Stress-CTP provides incremental therapeutic decision-making value compared to cCTA alone. A sequential strategy with cCTA+FFR_CT_+Stress-CTP is associated with the best compromise in terms of clinical impact and radiation exposure.

## Figures and Tables

**Table 1:** Study population characteristics.

**Baseline Characteristics**	
Number, n	291
Age, years (mean ± SD)	65 ± 9
Male, n (%)	222 (76)
BMI, kg/m^2^ (mean ± SD)	26.7 ± 4.2
**Risk Factors**	
Hypertension, n (%)	214 (74)
Smoker, n (%)	93 (32)
Hyperlipidaemia, n (%)	189 (65)
Diabetes, n (%)	55 (19)
Family History, n (%)	179 (62)
**Symptoms**	
Typical angina, n (%)	218 (75)
Atypical angina, n (%)	73 (25)
**Pre-test likelihood of CAD, % (mean ± SD)**	65 ± 15
**Reasons for invasive coronary angiography**	
Symptoms, n (%)	147 (50)
Positive exercise-EKG, n (%)	79 (27)
Positive stress echocardiography, n (%)	11 (4)
Positive single photon emission computed tomography, n (%)	49 (17)
Positive stress cardiac magnetic resonance, n (%)	5 (2)
**cCTA scan protocol, REST**	
HR before scanning, bpm (mean ± SD)	69 ± 11
β-blocker, n (%)	161 (55)
β-blocker dosage, mg (mean ± SD)	10 ± 5
HR during scanning, bpm (mean ± SD)	62 ± 9
Dose length product, mGy-cm (mean ± SD)	204 ± 92
Effective dose, mSv (mean ± SD)	2.9 ± 1.3
**cCTA scan protocol, STRESS**	
HR during scanning, bpm (mean ± SD)	81 ± 15
Effective dose for Static CTP, mSv (mean ± SD)	2.6 ± 1.0
Effective dose for Dynamic CTP, mSv (mean ± SD)	5.4 ± 0.6
**Prevalence of obstructive CAD (≥50%) at ICA**	
No disease, *n* (%)	97 (33)
One-vessel disease, *n* (%)	98 (34)
Two-vessels disease, *n* (%)	50 (17)
Three-vessels disease, *n* (%)	46 (16)
Prevalence of functionally significant CAD *	143 (49)

SD: standard deviation; BMI: body mass index; CAD: coronary artery disease; EKG: electrocardiogram; HR: heart rate; ICA: invasive coronary angiography: cCTA: coronary computed tomography angiography; CTP: computed tomography perfusion. *: >80% diameter reduction stenosis or fractional flow reserve (FFR) < 0.8 in intermediate stenosis (30–80% diameter reduction).

## Appendix B

**Rest cCTA performance and interpretation.** Rest cCTA was performed with a Revolution CT scanner (GE Healthcare, Milwaukee, Wisconsin). The following parameters were used: slice configuration 256 × 0.625 mm with scintillator detector, gantry rotation time 280 ms, tube voltage 120 KVp and 100 KVp in patients with body mass index >30 kg/m^2^ and <30 kg/m^2^, respectively, and an effective tube current of 500 mA. One beat axial scan was used in all patients with variable padding ranging from 70% to 80% and 40% to 80% of the cardiac cycle in patients with HR <65 beats/min and >65 beats/min, respectively. All patients received a 70-mL bolus of iodixanol 320 (Visipaque 320 mg/mL, GE Healthcare, Oslo, Norway) at an infusion rate of 6.2 mL/s followed by 50 mL of saline solution at the same rate of infusion. Datasets for each cCTA examination were transferred to an image-processing workstation (Advantage Workstation 4.7; GE Healthcare, Milwaukee, Wisconsin) and independently analyzed according to the Society of Cardiovascular Computed Tomography (SCCT) guidelines for reporting by two cardiac imagers (M.G. and G.M.) who were blinded to the clinical history of the patients. Coronary arteries were segmented as suggested by the American Heart Association. Impaired image quality was classified according to the Likert score. All non-evaluable coronary artery segments were censored as positive. Obstructive CAD was defined as the presence of stenosis >50%. A third cardiac imager (A.I.G.) with more than 5 years’ experience in cCTA adjudicated the scores in cases of disagreement.

**FFR_CT_ performance.** All cCTA datasets were sent to HeartFlow (Redwood City, California) for FFR_CT_ analysis. In brief, for each patient a quantitative 3-dimensional anatomic model combining the aortic root and epicardial coronary arteries was generated from the cCTA dataset. Subsequently, coronary blood flow and pressure were computed under specific conditions simulating maximal hyperemia.

**Static Stress-CTP performance and interpretation.** Static Stress-CTP scans was performed after vasodilation induced by intravenous administration of adenosine (0.14 mg/kg/min over 4 min). At the end of the third minute of adenosine infusion, a single data sample was acquired during the first-pass enhancement of cCTA by using the same protocol described for rest-cCTA. All datasets of Static Stress-CTP were transferred to a dedicated image-processing workstation (Advantage Workstation Version 4.7, GE Healthcare), and two cardiac imagers (A.B. and G.P.) blinded to the clinical history, rest cCTA, and invasive evaluation findings, independently evaluated the reconstructed images. The myocardial wall was evaluated on short-axis (apical, mid, and basal slices) and long-axis views (2-, 3- and 4-chamber projections) with 4-mm-thick average multiplanar reformatted images in diastolic phases (70% and 80% of cardiac cycle). A narrow window width and level (350 W and 150 L) was used for perfusion defect evaluation. Each myocardial segment was correlated to the specific coronary territory according to a modified AHA classification as described by Cerci et al. Perfusion defects were defined as subendocardial hypo-enhancements encompassing ≥ 25% of transmural myocardial thickness within a specific coronary territory.

**Dynamic Stress-CTP performance and interpretation.** Similar to Static Stress-CTP, Dynamic Stress-CTP scans were performed after vasodilatation induced with an intravenous adenosine injection (0.14 mg/kg/min over 4 min). At the end of the third minute of adenosine injection, 0.7 mL/kg of iodixanol 320 (Visipaque 320 mg/mL; GE Healthcare, Oslo, Norway) at an infusion rate of 5 mL/s followed by 0.5 mL/kg of saline solution at an infusion rate of 5 mL/s was injected and, at the same time, the Stress-CTP acquisition was performed in free breathing. Three consecutive series of datasets were acquired that covered the entire heart volume. Multiple short-axis views of the left ventricle from the base to the apex corrected for breathing-related displacement were generated and endocardial and epicardial borders were selected. Accordingly, the change of attenuation in the myocardium over time was computed. For quantification of myocardial blood flow (MBF), the arterial input function (AIF) was sampled in the ascending aorta and the myocardial time/attenuation curves were coupled with the AIF using a hybrid deconvolution model. Finally, MBF was computed by dividing the convoluted maximal slope of the myocardial time-attenuation curve by the maximum AIF. MBF maps were then reconstructed as a stack of color-coded images with a slice thickness of 3.0 mm. Measurements of MBF were obtained from regions of interest of at least 0.5 cm^2^ positioned in a representative area of each myocardial segment according to a standard 16-segment model. Blinded adjudication was performed to verify co-registration of CTP-defined perfusion defects with culprit vessels as defined using cCTA by two cardiac imagers (A.B. and G.P.) with more than 5 years of experience. Perfusion defects were defined as the presence of MBF values less than 101 mL/100 gr/min within a specific coronary territory.

**ICA and invasive FFR performance and interpretation.** Certified interventional cardiologists performed ICA in all patients within 60 days after the cCTA examination. Coronary angiograms were analyzed at the clinical site by an interventional cardiologist who was blinded to cCTA and Stress-CTP findings. The severity of coronary stenosis was quantified in two orthogonal planes by identifying the minimum diameter and reference diameter for all stenosis, and the percentage of stenosis was derived. Functionally significant CAD was defined as the presence of coronary artery stenoses ≥ 80%, a totally occluded vessel, or stenoses with invasive FFR < 0.8.

**Radiation exposure.** The effective radiation dose (ED) was calculated by multiplying the dose-length product and a conversion coefficient for the chest (K = 0.014 mSv/mGy × cm). For ICA, ED was calculated by multiplying the dose area product by a conversion factor (K = 0.21 mSv/mGy × cm^2^) for lateral and posterior-anterior radiation exposure in the chest.
